# Correction of irregular and induced regular corneal astigmatism with toric IOL after posterior segment surgery: a case series

**DOI:** 10.1186/s12886-016-0397-8

**Published:** 2017-01-13

**Authors:** Bence L. Kolozsvári, Gergely Losonczy, Dorottya Pásztor, Mariann Fodor

**Affiliations:** Department of Ophthalmology, Faculty of Medicine, University of Debrecen, Nagyerdei krt. 98, H-4012 Debrecen, Hungary

**Keywords:** Toric IOL, Scleral buckling, Keratoconus, Vitrectomy

## Abstract

**Background:**

Toric intraocular lens (IOL) implantation can be an effective method for correcting corneal astigmatism in patients with vitreoretinal diseases and cataract. Our purpose is to report the outcome of toric IOL implantation in two cases - a patient with scleral-buckle-induced regular corneal astigmatism and a patient with keratoconus following pars plana vitrectomy. As far as we are aware, there are no reported cases of toric IOL implantation in a vitrectomized eye with keratoconus nor of toric IOL implantation in patients with scleral-buckle-induced regular corneal astigmatism.

**Case presentation:**

Two patients with myopia and high corneal astigmatism underwent cataract operation with toric IOL implantation after posterior segment surgery. Myopia and high astigmatism (>2.5 diopter) were caused by previous scleral buckling in one case and by keratoconus in the other case. Pre- and postoperative examinations during the follow-up of included uncorrected and spectacle corrected distance visual acuity (UCDVA/CDVA), automated kerato-refractometry (Topcon), Pentacam HR, IOL Master (Zeiss) axial length measurements and fundus optical coherence tomography (Zeiss). One year postoperatively, the UCDVA and CDVA were 20/25 and 20/20 in both cases, respectively. The absolute residual refractive astigmatism was 1.0 and 0.75 Diopters, respectively. The IOL rotation was within 3° in both eyes, therefore IOL repositioning was not necessary. Complications were not observed in our cases.

**Conclusion:**

These cases demonstrate that toric IOL implantation is a predictable and safe method for the correction of high corneal astigmatism in complicated cases with different origins. Irregular corneal astigmatism in keratoconus or scleral-buckle-induced regular astigmatisms can be equally well corrected with the use of toric IOL during cataract surgery. Previous scleral buckling or pars plana vitrectomy seem to have no impact on the success of the toric IOL implantation, even in keratoconus. IOL rotational stability and refractive predictability in patients with a previous vitreoretinal surgery can be as good as in uncomplicated cases.

## Background

Cataract surgery is one of the most frequent surgeries worldwide. With the improvement of biometry procedures, including corneal topography and tomography, toric intraocular lens (IOL) implantation has become more and more predictable in correcting astigmatism [1–3].

The improvement in vitreoretinal surgical techniques has enabled eye surgeons to manage more challenging cases. However, vision rehabilitation can be cumbersome in patients with multiple refractive comorbidities. Toric IOL implantation can be an effective method for correcting corneal astigmatism in patients with vitreoretinal diseases and cataract. This is because toric IOLs show good rotational stability, even in vitrectomized eyes [2]. Cataract surgery with the implantation of toric IOL in stable keratoconus is a safe and effective procedure regarding keratometry stability, visual and refractive results [3–5]. As far as we are aware, there is no reported case of toric IOL implantation in a vitrectomized eye with keratoconus.

Scleral buckling is a great surgical procedure to treat rhegmatogenous retinal detachment but can induce permanent changes in the anatomy of both the anterior and posterior segments. As a result, myopic shift and high astigmatism frequently occur [6–8]. A unilateral difference in refraction creates anisometropia, and the correction with spectacle may be impossible. Different types of corneal laser refractive surgeries and phakic intraocular lenses implantations have been proved to be safe and predictable methods for reducing these refractive errors [6–8]. If cataract surgery is indicated, phacoemulsification with toric IOL implantation might give an opportunity to retain visual balance and may reverse the refractive status. To the best of our knowledge, no cases of toric IOL implantation in patients with scleral-buckle-induced regular corneal astigmatism have been reported before.

The purpose of this case series was to evaluate the long-term outcome of toric IOL implantation in a patient with irregular cornea astigmatism due to keratoconus following a pars plana vitrectomy and in a different patient with regular corneal astigmatism, caused by a previous scleral buckling procedure.

## Case presentation

This noncomparative, interventional case series was performed at a single center (Department of Ophthalmology, University of Debrecen) and all of the surgeries were performed by the same surgeon (M.F.). We enrolled two female patients with previous posterior segment surgeries and a postoperative corneal astigmatism that was greater than 2.5 diopters (D). In case 1, an encircling buckle and a radial sponge were placed to treat a rhegmatogenous retinal detachment. In case 2, a patient with keratoconus developed a vitreous hemorrhage, which was caused by a branch retinal artery occlusion. This was managed with 23-gauge vitrectomy. After these primary standard operations, the patients developed cataract, associated with myopia and high astigmatism.

In case 1, a previous scleral buckling procedure induced high regular corneal astigmatism. In accordance with the 70-year-old patient, we did not remove the encircling buckle or the radial sponge and neither did we choose excimer laser to treat the corneal astigmatism. Instead, to reduce the chance of possible complications, we chose cataract surgery and toric IOL implantation. This addressed both the cataract and high corneal astigmatism in a single session. In case 2, the cause of refractive error was a stage 1 keratoconus (based on Amsler-Krumeich Classification) stable for many years, as shown by Pentacam examinations. Cataract surgery with toric IOL implantation was offered for the 55-year-old patient, irrespective of the previous vitrectomy. Following a comprehensive discussion of the toric IOL implantation and potential risks, both of the patients gave written consent. A complete ophthalmological and medical history was achieved from both of the patients.

Both eyes underwent a standard phacoemulsification and toric IOL (case 1: +17.5D AcrySof Toric SN6AT7, case 2: +15.0 D AcrySof Toric SN6AT6) implantation using Infinity Vision System (Alcon Laboratories) through a 2.6 mm corneal incision. The preoperative and postoperative evaluations included an assessment of the uncorrected distance visual acuity (UCDVA), spectacle corrected distance visual acuity (CDVA), automated kerato-refractometry (KR-8900; Topcon Co, Tokyo, Japan), slitlamp biomicroscopy, indirect ophthalmoscopy and applanation tonometry. Both of the patients had Rotating Scheimpflug tomography (Pentacam HR, Oculus Optikgeräte GmbH, Wetzlar, Germany) and IOL Master (Carl Zeiss Meditec, Jena, Germany) before the toric IOL implantation and during the 12-month follow-up. Fundus OCT was used to exclude any fundus anomalies (i.e., astigmatism) in case 1.

The IOL powers were calculated using the SRK/T formula, and the targeted refraction was emmetropia. The IOL cylinder power and alignment axis were calculated using the web-based toric IOL calculator program (available at: www.acrysoftoriccalculator.com). The final IOL diopters were determined based on keratometry readings (kerato-refractometry, Pentacam HR, IOL Master) and axial length measurements (IOL Master). The position of the cylindrical axis of the IOL was preoperatively marked in a sitting position and postoperatively measured using a slit lamp (Haag-Streit, BC 900, Switzerland) with a thin coaxial rotated slit light.

The clinical data of the patients are summarized in Table 1. No patients had intraoperative or postoperative complications. The fundus OCT showed normal macular anatomy and no fundus astigmatism in case 1. No patients developed retinal detachment or vitreoretinal changes in the treated eye during the 12-month follow-up.

**Table 1:** Clinical data of patients along with the IOL design performed with 3 different devices

	Patient 1.	Patient 2.
Preoperative clinical data		
Vitreoretinal procedure; date of intervention; age at the operation	scleral buckling on the right eye (encircling buckle and a radial sponge); September 2011; 67 years old	23-gauge vitrectomy on the left eye; December 2013; 54 years old
Cause of refractive errors	scleral buckling implants (Figs. 1 and 2)	Keratoconus
UCDVA	20/200	20/500
CDVA	20/50 with −6.0 + 4.5 x 20 degrees	20/50 with −10.5 + 4.0 x 15 degrees
Preoperative measurements and IOL design		
Topcon KR8100 keratorefractometer	K1: 42.75D / 125° K2: 46.25D / 35° Calculated IOL type: AcrySof Toric T7	K1: 48.25D / 40° K2: 50.75D / 130° Calculated IOL type: AcrySof Toric T6
Pentacam HR	K1: 42.1D / 129° K2:45.3D /39° (Fig. 1) Calculated IOL type: AcrySof Toric T7	K1: 48.0 D / 45° K2: 50.2 D / 135° Calculated IOL type: AcrySof Toric T6
Zeiss IOL Master	K1: 43.05D / 127° K2: 45.98 D / 37° Calculated IOL type: AcrySof Toric T6	K1: 47.87 D / 46° K2: 50.60 D / 136° Calculated IOL type: AcrySof Toric T6
Axial length (mm) Zeiss IOL Master	24.47 mm	23.92 mm
Implanted toric IOL	+17.5D AcrySof Toric SN6AT7	+15.0 D AcrySof Toric SN6AT6
Axis of IOL positioning	35°	44°
Time of phacoemulsification	February 2014	June 2014
Postoperative measurements at 1 month		
Topcon KR8100 keratorefractometer	K1: 42.25 D 125° K2: 46.25 D 35°	K1: 47.5 D 30° K2: 50.75 D 120°
Pentacam HR	K1: 41.6 D 136° K2: 45.5 D 46°	K1: 47.4 D 37° K2: 50.0 D 127°
UCDVA	20/25	20/30
CDVA	20/20 with −1.0 x 120 degrees	20/20 with −0.5 −0.75 x 95 degrees
Postoperative measurements at 12 months		
Topcon KR8100 keratorefractometer	K1: 42.25 D K2: 46.25 D S: -0.5D / C: +1.0D/ SE: 0.0D	K1: 48.0 D K2: 50.75 D S: -2.0 / C: +0.75D/ SE: -1.75 D
Pentacam HR	K1: 41.6 D K2: 45.4 D	K1: 47.7 D K2: 50.5 D
UCDVA	20/25	20/20
CDVA	20/20 with - 0.5 x 115 degrees	20/20

*Abbreviations*: *IOL* intraocular lens, *K1/K2* keratometry values for the steep and flat axis, *AL* axial length, *UCDVA* uncorrected distance visual acuity, *CDVA* corrected distance visual acuity, AcrySof Toric SN6AT7, 4.5 D at IOL plane; AcrySof Toric SN6AT6, 3.75 D at IOL plane; *S* spherical (Diopter), *C* cylindric (Diopter), *SE* spherical equivalent (Diopter)

One year after the toric IOL implantation, the UCDVA was above 20/25 and the CDVA was 20/20 in both cases. The absolute residual refractive cylinders were 1.0 and 0.75 Diopters. The IOL rotational misalignment was within 3° in both eyes. No eyes required further IOL repositioning. Taken together, a significant improvement in CDVA and UCDVA was observed in both cases. Refraction was stable during the follow-up period (Table 1). Both of the patients were satisfied with their postoperative visual outcome.

## Conclusion

There are many surgical options for treating preexisting or induced corneal astigmatism including excimer laser refractive procedures, limbal or corneal relaxing incisions and phakic IOLs. All of these treatments have limitations including the degree of treatable astigmatism and long-term instability [1]. In the present paper, we report the successful management of two cases with cataract and high astigmatism after posterior segment surgeries, due to scleral buckling or keratoconus. The patients underwent cataract extraction with toric IOL implantation. Both of the patients showed marked improvement in visual acuity and subjective refraction, while retinal pathologies remained stable. Therefore, phacoemulsification with toric IOL implantation seems to be an effective method for correcting high astigmatism in cataract patients after posterior segment surgeries and corneal pathologies.

Although there are several new effective techniques to correct astigmatism due to corneal ectasias, the correction of high astigmatism in cataract patients presenting with keratoconus continues to be challenging [3]. AcrySof toric IOL is considered to be one of the most commonly used toric IOL in cataract surgery with a handy and reliable on-line calculator. However, IOL power calculation is still a challenge in cataract patients with keratoconus [3]. Cataract surgery with implantation of toric IOL in stable keratoconus is a safe and effective procedure regarding keratometry stability, visual and refractive results [5]. The first result with toric IOL implantation after vitrectomy was reported in 2011, and showed that combined phacovitrectomy with toric IOL implantation is an effective method for correcting vitreoretinal diseases, cataract and pre-existing regular corneal astigmatism [1]. In vitrectomized eyes, the postoperative IOL axis stability for one year is similar to cataract surgery alone. Furthermore it has been proved that the vitrectomized status of eyes does not provoke mispositioning of toric IOL [2]. The similar incidence of rotation to the non-vitrectomized eyes may be derived from the special haptic design and adhesive properties of the AcrySof toric IOL [1, 2, 9]. Our keratoconus patient had cataract, which was induced by a previous vitrectomy. Since an accurate determination of K values and cylindrical axis is a prerequisite of a successful outcome, we used IOL Master and Pentacam HR for these measurements. These provided similar results including the cylindrical power of the toric IOL. To the best of our knowledge, there is no reported case of implantation a toric IOL in keratoconus after vitrectomy. Our results highlight that AcrySof toric IOL shows good rotational stability after vitrectomy, even in keratoconic eyes.

Scleral buckles indent the sclera, changing the sagittal dimensions of the globe and potentially causing corneal or fundus astigmatism [6–8]. In our patient, after scleral buckling surgery (encircling buckle and a radial sponge) we observed a myopic shift of 1.5 D, which is in-line with previous results [6, 8]. Besides, we observed an unusually high corneal astigmatism of 3.5 D (Figs. 1 and 2). One month and also 12 months after toric IOL implantation, our patient was within the ± 0.5 D of the planned correction. This result is better than those reported cohorts of patients after scleral buckling having corneal refractive surgery for correcting anisometropia [6]. Based on earlier studies, photorefractive keratectomy (PRK) seems to be more predictable in patients who have had previous scleral buckling surgery. However, the reason that the attempted result versus the achieved result is unfavorable in patients who have had refractive surgery after scleral buckling is unknown [6]. Calculating the cylindric power of the toric IOL the Pentacam HR and the IOL Master did not give similar results in our case, and we implanted the IOL correcting for the higher cylinder, measured by the Pentacam HR and the kerato-refractometer. The postoperative refractive results proved that this was a good decision. Further studies are needed to compare whether toric IOL implantation or corneal refractive surgery gives predictable results for anisometropia and astigmatism correction after scleral buckling surgery.

**Fig. 1:**
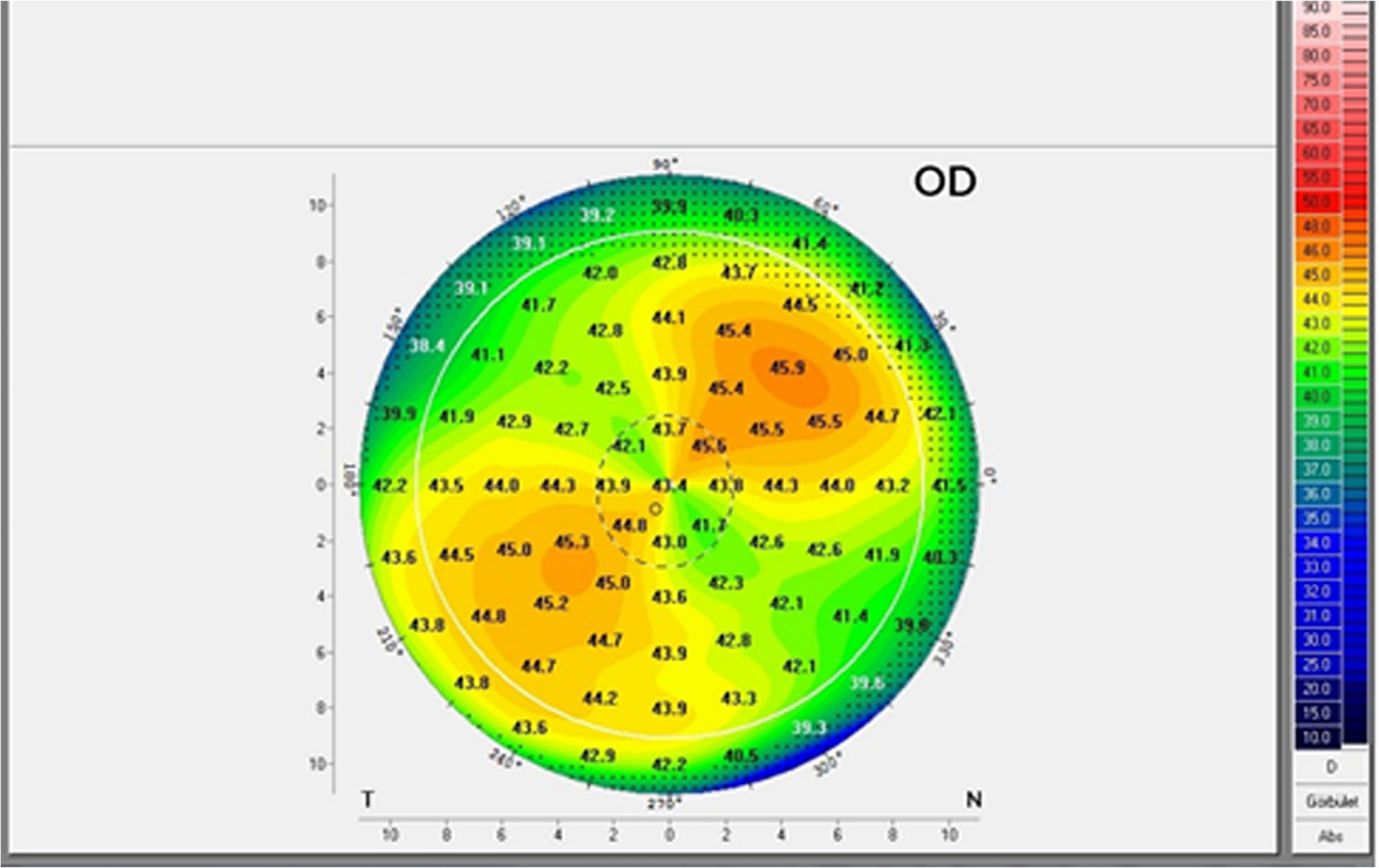
Preoperative topography with Pentacam HR. High regular astigmatism is detected caused by a radial sponge

**Fig. 2:**
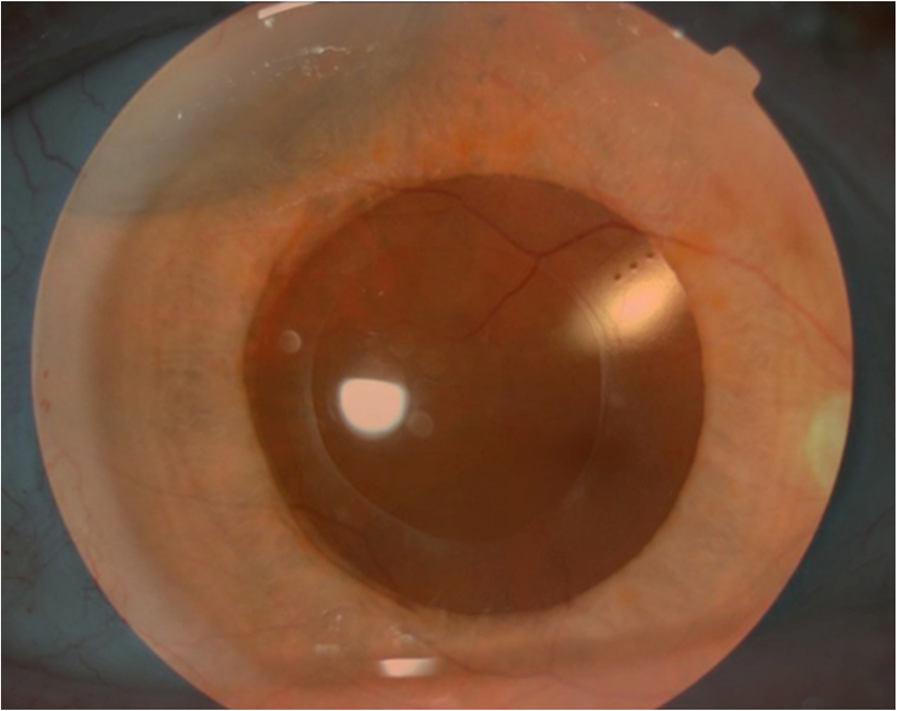
Postoperative montage photo. Postoperative montage photo with toric IOL (see at 2 o’clock the cylinder axis marks on the posterior surface of the IOL) and the superotemporal located radial sponge. The axis of the cylinder marks and the sponge is perpendicular

These cases demonstrate that toric IOL implantation may be safe and effective for the correction of refractive errors induced by retinal detachment repair or after vitrectomy in patients with corneal ectasia. The results of toric IOL implantation are always uncertain in previously none published complicated cases. The report of such cases broadens the spectrum of conditions in which toric IOLs can be used with certainty. Both of our cases were complicated but the patients were well educated and agreed to implantation of the toric IOL, despite the previous vitreoretinal surgery. They were satisfied with the postoperative visual outcomes.

Our study has several limitations. Due to the limited number of cases and absence of previous reports, it is hard to precisely demonstrate the efficacy of toric IOL in vitrectomized, keratoconic eyes, as well as in eyes with induced astigmatism caused by scleral buckling implants. However, this study shows that AcrySof toric IOL has good rotational stability, achieving optimal refractive and visual outcomes, even in vitrectomized keratoconic eyes. This is also the case in eyes with induced astigmatism caused by scleral buckling implants, which has not been reported before. Additionally, Pentacam is considered to be an accurate tool for obtaining K values and axis measurements, showing no differences compared with IOL Master in case of vitrectomized, keratoconic eyes. However, it could be said that Pentacam gives more precise calculation than IOL Master after scleral buckling surgery, and complimentary examinations are required for the decision (keratorefractometer, Orbscan).

In conclusion, after different types of posterior segment surgeries phacoemulsification with AcrySof, toric IOL implantation can be an effective method for correcting pre-existing irregular astigmatism resulting from ectatic corneal diseases, as well as surgically induced regular astigmatism. Further randomized and prospective studies with a larger study population are necessary to evaluate the efficacy, safety and predictability of toric IOLs, and our study could be a useful reference for these future trials. The reconsideration of the contraindication for toric IOL implantation seems to be relevant. Most importantly, this is because the quality of life of these patients with complicated problems can improve dramatically with toric IOL implantation. We believe that our case series provides novel and valuable information on the treatment of extraordinary cases. Moreover, it seems unlikely that toric IOL implantation would increase the risk for retinal detachment or other vitreoretinal complications after previous retinal surgery (scleral buckling or vitrectomy).

## Abbreviations

CDVA: Spectacle corrected distance visual acuity
D: Diopters
IOL: Intraocular lens
PRK: Photorefractive keratectomy
UCDVA: Uncorrected distance visual acuity
